# Editorial: Intestinal Inflammation

**DOI:** 10.3389/fmed.2018.00318

**Published:** 2018-11-13

**Authors:** Luca Pastorelli, Franco Scaldaferri

**Affiliations:** ^1^Gastroenterology Unit, IRCCS Policlinico San Donato, San Donato Milanese, Italy; ^2^Internal Medicine, Gastroenterology and Hepatic Diseases Unit, Gastroenterological Area, Gastroenterological and Endocrino-Metabolical Sciences Department, Fondazione Policlinico Universitario A. Gemelli, Università Cattolica del Sacro Cuore, Rome, Italy

**Keywords:** inflammatory bowel diseases, Crohn's disease, ulcerative colitis, coeliac disease, innate immunity, microbiota, exposome

The gastrointestinal tract represents the vastest surface of human body and, while carrying out its physiological task of digesting and selectively absorbing nutrients, it is constantly exposed to a huge variety of chemical challenges and, most importantly, to an enormous load of microorganisms, which dwells in the gut lumen.

Several mechanisms maintain the delicate balance between an efficient passage of nutrients across the gut mucosa and an effective immunological control over harmful microbial pathogens, while permitting the colonization, but not penetration, by normal gut microbiota (1). Indeed, temporary or persistent perturbations of the aforementioned equilibrium cause a disruption of intestinal homeostasis, leading to the onset of inappropriate immune and inflammatory responses, eventually becoming themselves the source of intestinal damage and malfunction, like in inflammatory bowel diseases (IBD), microscopic colitides, and coeliac disease (2, 3).

In this Research Topic, global experts in mucosal immunology address pivotal key points to consider for unraveling pathophysiological mechanisms of chronic intestinal inflammation.

Since the discovery of the association of polymorphisms of NOD2/CARD15 gene to Crohn's disease, researchers focused their efforts in understanding innate immunity in the gut, generally reaching the conclusion that defects in this primary branch of immunity may be a very early event in disrupting gut homeostasis (2). The article by Corridoni et al. provides a current comprehensive overview on intestinal innate immunity and mucosal inflammation and discusses novel therapeutic approaches for IBD, exploiting the regulation of innate immunity to dampen gut inflammation, in experimental murine and human colitides. Indeed, we are all waiting for the results of these trials and, hopefully, in few years we will see a widening of our therapeutic tools for IBD.

Deeply connected with innate immunity is the presence, composition and interactions of gut resident microorganisms, constituting host intestinal microbiota. In fact, it is becoming more and more evident how gut microflora can shape intestinal immune responses and specific microbiota profiles can elicit inflammation whereas other possess the ability to dampen it down (4). Indeed, manipulations of gut microbiologic environment results in modifications of host immunological assets. Consistently, the paper from Burrello et al. aims to explore, in a murine model of colitis, the effect of a wide spectrum antibiotic treatment on the activation of invariant natural killer T cells, a subset of immune cells highly reactive to changes in gut microbiota composition. Following the antibiotic treatment, the onset of intestinal dysbiosis triggers changes in activation and phenotype of those cells, which become pro-inflammatory, producing greater amount of interferon-γ and inteleukin-17A; interestingly, those changes are reversible over time, but can become sustained if microbiota alterations are prolonged by dysbiotic fecal material transplant. As a matter of fact, these data also suggest the therapeutic potential of interventions aimed to manipulate intestinal bacterial content. This is in line with the manuscript from Vecchione et al. which further stresses the importance of selecting the proper probiotic compound, in order to obtain a significantly beneficial effect on gut homeostasis. This article analyzes different commercially available probiotic formulations, taking into account several features and parameters which should assure the effectiveness of the compounds. Actually, researchers and clinicians should consider those characteristics when selecting appropriate probiotic compound to prescribe. In recent years, aside from microbiota modulation, other therapeutic approaches alternative to classic direct immunomodulation have been proposed to manage immune disorders of the gut; among these, antigen-based immunotherapy, a specific immunologic approach aimed to restore tolerance to definite antigens has been proposed also in the field of mucosal immunology (5); more in details, phase I trials have been performed in order to test this particular strategy in coeliac disease. The review from Di Sabatino et al. summarize available information on this topic, providing a comprehensive analysis of the data; indeed, given the unique immunological features of coeliac disease, this approach is based on a solid rationale; however, in the next future, a better understanding of immunopathologic events leading to the onset of other intestinal inflammatory diseases might deliver theoretical basis to test antigen desensitization in a broader array of gut disorders.

As a matter of fact, at present time, despite the huge expansion in knowledge witnessed in the last decades, we are still far from fully comprehend the mechanisms underpinning the inception and perpetration of intestinal inflammation in human disease; one of the biggest obstacles that needs to be overcome is the overwhelming complexity of the system: inflammatory conditions of the gut originate from multidirectional interactions between environmental conditions, host genetics, epigenetic regulation, immune system features and intestinal microbiota composition. So far, the scientific approach tended to dissect every single mechanism, providing detailed notions on single biological events; however, in order to move forward, we also need to integrate the huge amount of data we have and consider the whole phenomenon in its integrity (6). This issue is covered and critically discussed in the insightful opinion article from Fiocchi; he suggests that system biology and network medicine, may be the disciplines that will help us to overcome complexity and variability in order to integrate all available knowledge on the matter.

Overall, we are very pleased of having worked on this Research Topic, as all the Authors provided outstanding contributions which, we hope, will grant readers novel prospects and stimuli for their research in gut mucosal immunology.

## Author contributions

LP and FS conceived and wrote the manuscript. All the authors approved the final version of the manuscript and fully agreed with its content.

### Conflict of interest statement

The authors declare that the research was conducted in the absence of any commercial or financial relationships that could be construed as a potential conflict of interest.
